# Surgical Treatment for Spinal Scoliosis in Patient With Mitochondrial Disease After Heart Transplantation: A Case Report and Literature Review

**DOI:** 10.1155/cro/8814759

**Published:** 2026-03-04

**Authors:** Shintaro Abe, Masayuki Miyagi, Wataru Saito, Ryo Tamaki, Tomohisa Inoue, Kenji Hagiwara, Gen Inoue, Ken Okazaki, Masashi Takaso

**Affiliations:** ^1^ Department of Orthopaedic Surgery, School of Medicine, Kitasato University, Sagamihara, Kanagawa, Japan, kitasato-u.ac.jp; ^2^ Department of Orthopaedic Surgery, School of Medicine, Tokyo Women’s Medical University, Tokyo, Japan, twmu.ac.jp

**Keywords:** heart transplantation, mitochondrial disease, posterior spinal correction and fusion surgery, scoliosis

## Abstract

**Background:**

We present an extremely rare case of scoliosis surgery for mitochondrial disease after heart transplantation.

**Case Presentation:**

An 18‐year‐old female patient with mitochondrial disease underwent heart transplantation for dilated cardiomyopathy at age 12. After heart transplantation, she presented with progressive scoliosis with a Cobb angle of 101°. Therefore, we performed spinal correction and fusion surgery under strict management. Postoperative spinal radiography revealed successful spinal correction and maintained correction 2 years after surgery.

**Conclusions:**

This is the first report of scoliosis surgery in a patient with mitochondrial disease after heart transplantation. Scoliosis surgery in patients after heart transplantation requires strict management of the circulating intravascular volume, because the denervated heart is preload‐dependent. Moreover, patients with mitochondrial disease should be treated for lactic acidosis caused by surgical stress and perioperative electrolyte abnormalities. Our case showed successful performance in minimizing invasiveness without compromising the surgical goals of correction and fusion.

## 1. Introduction

Mitochondria play a critical role in energy metabolism. Mitochondrial diseases are disorders caused by mitochondrial dysfunction. Patients with mitochondrial diseases show a variety of symptoms, including seizures, epilepsy, migraines, psychiatric symptoms, muscle weakness, diabetes, and cardiomyopathy [1]. Muscle weakness has sometimes been reported to present with progressive scoliosis [2]. Moreover, heart transplantation is associated with severe cardiomyopathy [3, 4]. However, there are very few reports on the surgical treatment for scoliosis in post–heart transplant patients or in patients with mitochondrial disease. Here, we present an extremely rare case of scoliosis surgery for mitochondrial disease after heart transplantation and present a review of the literature.

## 2. Report of the Case

**Figure 1:**
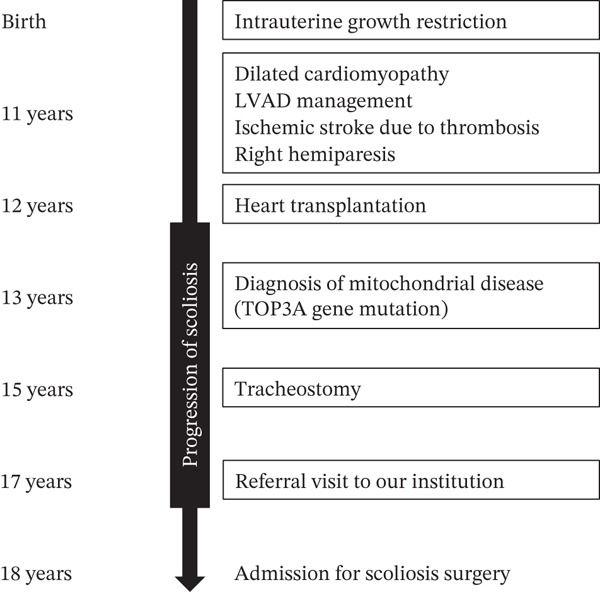
Clinical timeline of the patient. This timeline summarizes the patient’s clinical course from birth to surgical treatment for scoliosis.

The patient was an 18‐year‐old female. The patient was born with intrauterine growth retardation. At the age of 11 years, she was diagnosed with dilated cardiomyopathy. During a procedure to exchange a left ventricular assist device as part of her medical management, she developed an ischemic stroke caused by thrombosis, resulting in right‐sided hemiparesis. At 12 years of age, she underwent orthotopic heart transplantation for end‐stage dilated cardiomyopathy. At 13 years of age, a skin biopsy was performed, and she was diagnosed with mitochondrial disease associated with a TOP3A gene mutation. At 15 years of age, she required tracheostomy due to severe pneumonia. Following heart transplantation, she was diagnosed with scoliosis, which showed progressive worsening over time. At 17 years of age, she was referred to our hospital for further evaluation and management of the spinal deformity. At 18 years of age, she was admitted to our hospital with the aim of undergoing surgical correction of scoliosis. A timeline flowchart summarizing the sequence of clinical events from birth to surgery is shown in Figure 1.

On initial assessment at administration for scoliosis surgery, she was 138.0 cm tall and weighed 28.1 kg, with a short stature and low body weight. She received tube feeding and was able to maintain a standing position but usually used a wheelchair. Preoperative electrocardiography revealed a heart rate of 116 beats/min, sinus rhythm, and sinus tachycardia. Echocardiography revealed an ejection fraction of 71%, and the transplanted heart functioned well. Pulmonary function tests could not be performed because the patient had undergone tracheotomy; however, she was diagnosed with restrictive ventilatory impairment before tracheotomy.

**Figure 2:**
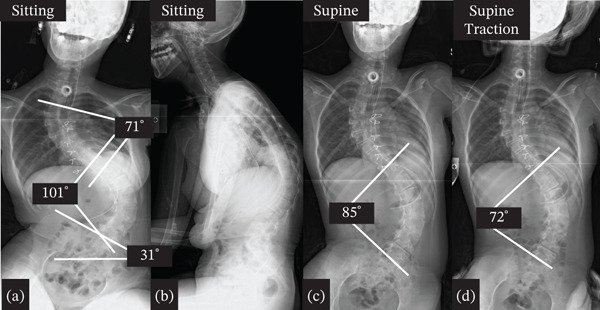
Preoperative x‐ray. (a) Anteroposterior (AP) view of sitting full spine, (b) lateral view of sitting full spine, (c) AP view of the supine full spine, and (d) AP view of the full spine in traction. The Cobb angle was measured between the upper endplate of the most tilted vertebra at the upper end and the lower endplate of the most tilted vertebra at the lower end of the curve. Pelvic obliquity was defined as the angle between a horizontal reference line and the line connecting the highest points of the bilateral iliac crests. All measurements were performed using EV Insite (PSP Corporation, Tokyo, Japan).

**Figure 3:**
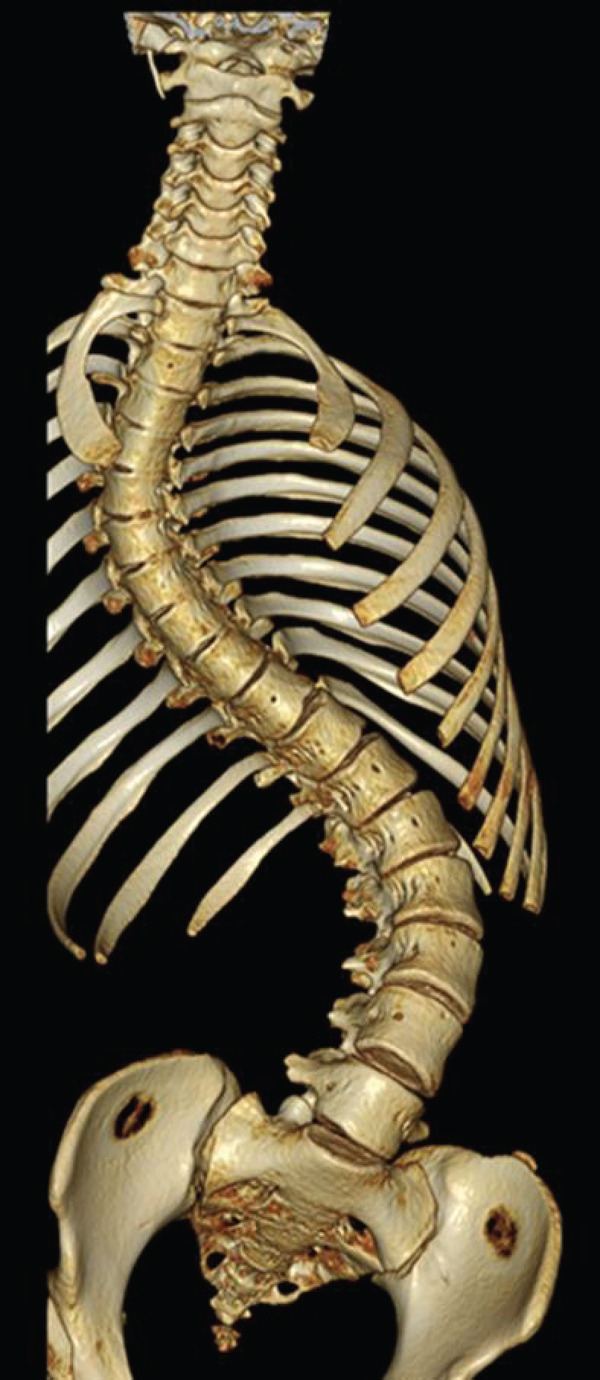
Preoperative three‐dimensional computed tomography of the full spine.

Preoperative sitting whole‐spine frontal radiography showed severe scoliosis with a Cobb angle of the upper thoracic curve from thoracic (T)3 to T11 of 71°, Cobb angle of the main thoracic curve from T11 to lumbar (L)5 of 101°, and pelvic obliquity of 31° (Figure 2a,b). The Cobb angle of the main thoracic curve from T11 to L5 in the supine position was 85° with a correction rate of 16% (Figure 2c). The Cobb angle of the main curve from T11 to L5 was 72° in the supine and traction positions, with a correction rate of 29% (Figure 2d). Preoperative three‐dimensional computed tomography of the entire spine revealed no abnormalities in the vertebral body (Figure 3). On the basis of these findings, the patient was diagnosed with symptomatic scoliosis associated with mitochondrial dysfunction.

**Figure 4:**
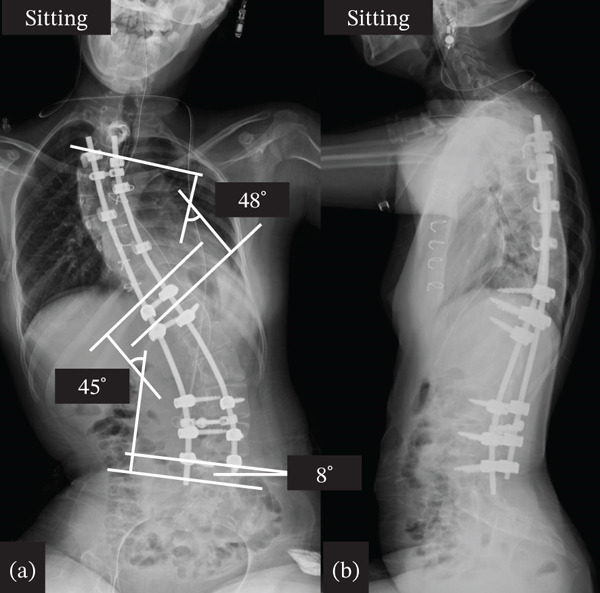
Postoperative x‐ray. (a) Anteroposterior view of sitting full spine. (b) Lateral view of sitting full spine.

**Figure 5:**
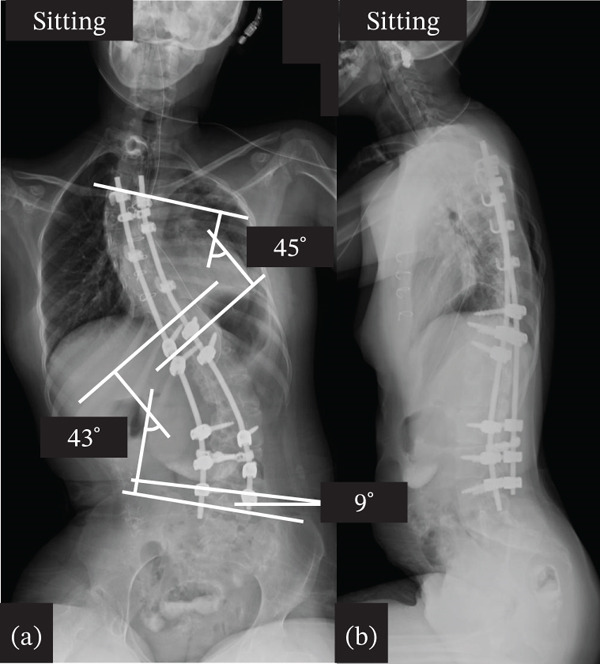
Postoperative x‐ray at the final follow‐up. (a) Anteroposterior view of sitting full spine. (b) Lateral view of sitting full spine.

Therefore, we performed spinal correction and fusion surgery under strict management by anesthesiologists and pediatricians. We made a skin incision at the midline of the back and widely exposed the spinal structure from the upper thoracic spine to the L5 level. After removing the soft tissue, pedicle screw instrumentation was placed bilaterally at T11, T12, L3, L4, and L5 using O‐arm navigation. Hooks were installed at the proximal levels, and sublaminar cables (Nesplon Cable System, Alfresa, Tokyo, Japan) were installed at the proximal and apex levels. Additionally, we performed facetectomy at several apical levels to obtain spinal flexibility. Surgery to correct the spinal deformity was performed by combining a rod rotation and cantilever technique, as previously reported [5]. Finally, we decorticated and placed a local autograft mixed with a bioresorbable bone graft. In this case, intraoperative neuromonitoring was utilized during the correction procedure. Anesthesia was maintained with total intravenous anesthesia using remifentanil and fentanyl. For the management of intraoperative blood loss, we used a cell saver and a radiofrequency bipolar sealer (Aquamantys, Medtronic, Minneapolis, Minnesota, United States). The operative time was 3 h 17 min, and blood loss was 276 mL. Postoperative spinal radiography revealed successful spinal correction, with a Cobb angle of the upper thoracic curve of 48° and a Cobb angle of the main thoracic curve of 45° (Figure 4). After surgery, strict management in the pediatric intensive care unit was required, and the patient was discharged 25 days after surgery. Prior to surgery, the patient expressed difficulty maintaining a stable sitting balance due to discomfort and fatigue. After the procedure, the patient reported improved sitting balance and overall comfort in daily activities, noting that these symptoms had significantly eased. At the final follow‐up 5 years after surgery, radiographs demonstrated sustained correction of the spinal deformity, and the patient continued to maintain a stable sitting balance while maintaining general condition, including cardiac and pulmonary function (Figure 5).

## 3. Discussion

Cases of scoliosis surgery with mitochondrial disease after heart transplantation are extremely rare. Only three cases in two papers were previously published concerning scoliosis surgery for mitochondrial disease [2, 6]. The incidence of scoliosis in patients with mitochondrial disease is approximately 5%, which is higher than that reported in the normal population [2]. Muscle weakness is the reported mechanism by which patients with mitochondrial disease present with progressive scoliosis [2]. On the other hand, there have been only two reports of spinal scoliosis surgery in patients after heart transplantation [7, 8]. This is the first report to present posterior spinal correction and fusion surgery for spinal scoliosis in a patient with mitochondrial disease after heart transplantation.

Regarding the relationship between scoliosis and heart disease, the incidence of scoliosis in patients with congenital heart disease has been reported as 4.5%–10.9%, which is higher than that in the normal population [9–11]. In particular, patients with congenital heart disease who underwent chest surgery showed a higher incidence of scoliosis, which was possibly associated with chest surgery [10]. In the present case, progressive scoliosis was observed after heart transplantation; therefore, heart transplantation and mitochondrial disease were considered associated with progressive scoliosis.

For the perioperative management of patients with mitochondrial disease, it has been reported that surgical stress, including hypothermia, hypoglycemia, hypovolemia, hypoxia‐induced lactic acidosis, and perioperative electrolyte abnormality, should be minimized [6, 12]. In addition, propofol has been reported to decrease mitochondrial function [13]. Thus, selecting an appropriate anesthetic agent is important for surgery in patients with mitochondrial diseases. For the perioperative management of patients after heart transplantation, the denervated heart is reported to have difficulty regulating the autonomic nervous system and to be preload dependent [7, 8]. Moreover, immunosuppressive agents, which are usually administered to patients undergoing heart transplantation, may delay wound healing and surgical site infection [7, 8]. These findings indicate that surgical stress should be minimized, and attention should be paid to postoperative complications, including delayed wound healing and infection. Therefore, we successfully performed surgery for severe scoliosis under strict management by anesthesiologists and pediatricians.

To minimize surgical stress, it is necessary to decrease the operating time and blood loss. Previous reports have demonstrated that the amount of blood loss and bleeding speed in the release stage of scoliosis surgery are higher than those in other stages of surgery [14–16]. In the present case, only a lower facetectomy and not Ponte osteotomy was performed during the release stage. Thus, our case showed successful performance in minimizing invasiveness by decreasing the operative time and blood loss without compromising the surgical goals of correction and fusion.

In conclusion, we present an extremely rare case of scoliosis surgery in a patient with mitochondrial disease after heart transplantation and a review of the literature. We were able to perform the surgery safely by minimizing invasiveness, including decreasing operative time and blood loss, under strict management by anesthesiologists and pediatricians.

## Author Contributions

Shintaro Abe drafted the manuscript. Masayuki Miyagi, Wataru Saito, and Masashi Takaso participated in surgery. Ryo Tamaki, Tomohisa Inoue, Kenji Hagiwara, and Ken Okazaki were responsible for postoperative management. Gen Inoue revised the manuscript.

## Funding

No funding was received for this manuscript.

## Ethics Statement

Ethics approval was excused for this study. The patient and the family were informed that the data from the research would be submitted for publication, and their written consent was obtained.

## Conflicts of Interest

The authors declare no conflicts of interest.

## Data Availability Statement

The data that support the findings of this study are available on request from the corresponding author. The data are not publicly available due to privacy or ethical restrictions.
